# Self-organized criticality in structured neural networks

**DOI:** 10.1186/1471-2202-14-S1-P168

**Published:** 2013-07-08

**Authors:** Maximilian Uhlig, Anna Levina, Theo Geisel, Michael J Herrmann

**Affiliations:** 1 Bernstein Center for Computational Neuroscience Göttingen, 37077, Germany; 2 MPI for Dynamics and Self-Organization, Am Faßberg 17, 37077 Göttingen, Germany; 3 Max-Planck-Institute for Mathematics in the Sciences, Leipzig, 04103, Germany; 4 University of Edinburgh, IPAB, School of Informatics, Edinburgh EH8 9AB, U.K

## 

Critical dynamics in neural networks is an experimentally and conceptually established phenomenon which has been shown to be important for information processing in the brain. Critical neural networks have been shown to have optimal computational capabilities, information transmission and capacity [1,2]. At the same time the theoretical understanding of neural avalanches has been developed starting from sandpile-like system and homogeneous networks towards structured networks. The network connectivity has been chosen, however, as to support or even to enable criticality. There are, nevertheless, many influences that shape the connectivity structure and weighting. Most prominently, this includes Hebbian learning and homeostatic effects, but also pathological changes.

We study how the structural changes affect the presence of criticality in the networks. While homeostatic plasticity may well have a regulatory effect that supports criticality, this cannot been said about Hebbian learning which essentially imprints structure from internally or externally caused activation patterns in the synaptic weighting of the network increasing thus the probability of previous patterns to reoccur. Unless the patterns are carefully chosen to produce critical behavior, these effects have a tendency to counteract critical behavior, e.g. by introducing a particular scale that corrupts the power-law distributions characteristic for critical behavior. Little is known, in particular, about the influence of criticality on associative memory neural networks.

We found that the critical regime is can be stabilized by short-term synaptic dynamics in the form of synaptic depression and facilitation that was already shown to play an important role in the self-organization of critical neural dynamics [3] or, alternatively, by homeostatic adaptation of the synaptic weights. We show that a heterogeneous distribution of maximal synaptic strengths does not preclude criticality if the Hebbian learning is alternated with periods of critical dynamics recovery.

**Figure 1:**
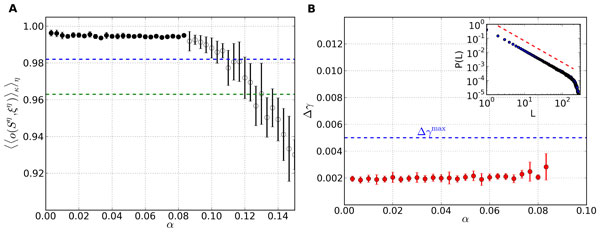
**A: Retrieval performance for networks with dynamical synapses and subject to Hebbian learning for different load parameters α**. Shown is the average overlap between stored patterns and the corresponding retrieved patterns. Dashed lines indicate overlaps corresponding to an average distortion by two digits (lower line) and one digit (upper line). **B: **Average mean-squared deviation Δγ from the best-fit power law. All data points lie below the threshold of Δγ = 0.005 for critical distribution. The inset shows an example avalanche size distribution *P*(*L*) in the converged state and the red dashed line marks the slope of the best-fit power law.

## References

[B1] Beggs J Plenz D Neuronal avalanches in neocortical circuits J Neurosci 2003 23 11167 11177 14657176 10.1523/JNEUROSCI.23-35-11167.2003 PMC6741045

[B2] Shew WL Plenz D The functional benefits of criticality in the cortex Neuroscientist 2013 19 88 100 10.1177/1073858412445487 22627091

[B3] Levina A Herrmann JM Geisel T Dynamical synapses causing self-organized criticality in neural networks Nat Phys 2007 3 857 860 10.1038/nphys758

